# Study protocol: MRI-based assessment of cerebral blood flow under pharmacologically elevated blood pressure in patients under general anesthesia, and in sedated ICU patients with aneurysmal subarachnoid hemorrhage

**DOI:** 10.1371/journal.pone.0338688

**Published:** 2025-12-10

**Authors:** Jonas Österlind, Johan Birnefeld, Elin Birnefeld, Magnus Hultin, Sara Qvarlander, Anders Wåhlin, Petter Holmlund, Laleh Zarrinkoob

**Affiliations:** 1 Department of Diagnostics and Intervention, Anesthesiology and Intensive Care, Umeå University, Umeå, Sweden; 2 Department of Clinical Sciences, Neurosciences, Umeå University, Umeå, Sweden; 3 Department of Diagnostics and Intervention, Biomedical Engineering and Radiation Physics, Umeå University, Umeå, Sweden; 4 Department of Diagnostics and Intervention, Umeå Centre for Functional Brain Imaging (UFBI), and Department of Applied Physics and Electronics, Umeå University, Umeå, Sweden; 5 Department of Applied Physics and Electronics, Umeå University, Umeå, Sweden; PLOS: Public Library of Science, UNITED KINGDOM OF GREAT BRITAIN AND NORTHERN IRELAND

## Abstract

**Background:**

Maintaining cerebral perfusion during anesthesia and intensive care is critical, yet the relationship between mean arterial pressure (MAP) and cerebral blood flow (CBF) remains poorly defined. In patients with aneurysmal subarachnoid hemorrhage (aSAH), pharmacologically induced hypertension is commonly applied to support cerebral perfusion, but its effects are uncertain.

**Methods:**

This protocol describes two parallel clinical studies using identical methodology. The first study population includes adults undergoing elective general anesthesia (MAP-ANE), and the second comprises sedated intensive care patients with aSAH (MAP-SAH). In both study populations, MAP will be increased stepwise with norepinephrine (NE) infusion under continuous invasive blood pressure monitoring, and CBF measured with phase-contrast MRI (PCMRI) and arterial spin labeling (ASL), while near-infrared spectroscopy (NIRS) will be performed in parallel to evaluate its validity as a surrogate marker. The primary outcome is the change in total CBF between baseline and elevated MAP, directly testing whether induced hypertension increases CBF. Secondary outcomes include ASL perfusion changes, the slope of the MAP–CBF relationship, systemic–cerebral hemodynamic correlations, and NIRS responses.

**Expected impact:**

These studies test the hypothesis that pharmacological MAP augmentation does not predictably increase CBF. By combining quantitative MRI with invasive monitoring, it aims to clarify MAP–CBF interactions, define the physiological basis of induced hypertension, and assess whether NIRS can serve as a clinically useful proxy. Findings are expected to inform safer and more individualized blood pressure management in perioperative and neurocritical care. The studies are registered at ClinicalTrials.gov (MAP-ANE: NCT06855407; MAP-SAH: NCT06033378).

**Trial registration:**

ClinicalTrials.gov, MAP-ANE NCT06855407, MAP-SAH NCT06033378

## Introduction

Tailoring blood pressure management to surgical procedure, clinical context, and comorbidities reduces the risk of organ dysfunction [1], but no consensus exists on intraoperative target levels, and the thresholds that define pathological blood pressure remain debated. A mean arterial pressure (MAP) of ≥65 mmHg is commonly assumed to be sufficient for organ perfusion [2], but individual patients may require higher levels depending on comorbidities.

In awake patients, the level of consciousness can serve as an indirect indicator of cerebral perfusion. Under general anesthesia or in critically ill patients, however, this assessment becomes considerably more challenging. This underscores the importance of understanding cerebral autoregulation, often illustrated by the classical model of Lassen (1959), which depicts a plateau of relatively constant cerebral blood flow (CBF) across a wide MAP range of approximately 60–160 mmHg [3]. More recent evidence demonstrates significant interindividual variability and a narrower plateau than originally described [4]. These uncertainties are clinically important not only in anesthesia, where hypotension frequently occurs, but also in neurological emergencies, which may present with both hypo- and hypertension [5] One such emergency is aneurysmal subarachnoid hemorrhage (aSAH), a life-threatening stroke caused by rupture of an intracranial aneurysm with bleeding into the subarachnoid space and, in some cases, extension into the brain parenchyma [6]. After treatment of the initial hemorrhage with endovascular coiling or surgical clipping, patients remain at risk of delayed cerebral ischemia (DCI) and associated neurological deficits during the early phase. [7]. In this setting, induced hypertension, an established therapy involving increase in blood pressure to supra-normal levels, is used to support cerebral perfusion [8,9]. Despite its frequent use, the evidence is limited, its effects on CBF remain uncertain, and the therapy carries risk [10].

Norepinephrine (NE) is the first-line vasopressor for restoring blood pressure in perioperative and critical care settings [11–13] and is also commonly used to achieve induced hypertension [14]. However, experimental and clinical studies, most relying on transcranial Doppler (TCD) flow velocity as a surrogate for CBF, indicate that NE-induced MAP elevation can have variable cerebrovascular effects depending on autoregulatory status. This reliance on surrogate markers leaves the net impact of NE on CBF unresolved [15,16].

Despite advances in understanding cerebral pathophysiology, clinical practice still relies primarily on MAP or, when intracranial pressure (ICP) is monitored, cerebral perfusion pressure (CPP) [17,18]. Bedside techniques such as near-infrared spectroscopy (NIRS) offer non-invasive, continuous monitoring of regional cerebral oxygenation (rSO_2_) [19]. However, because NIRS reflects surrogate markers of blood flow rather than direct measurements, it carries a risk of misinterpretation, particularly given that the relationship between MAP and CBF has not been fully characterized in different clinical contexts. Direct quantitative methods are therefore required. Advanced MRI techniques, notably phase-contrast MRI (PCMRI) and arterial spin labeling (ASL), provide non-invasive, contrast-free quantification of CBF. PCMRI enables measurement of blood flow rate (mL/min), vessel diameter, pulsatility, and flow direction in large cerebral vessels and is widely regarded as the gold standard for non-invasive quantification [20]. ASL provides regional cerebral perfusion measurements in mL/min/100 g and is widely used in clinical and research contexts [21]. Combined, PCMRI and ASL allow complementary quantification of CBF. When integrated with MAP and systemic circulation measures, this approach can yield valuable insights into the interaction between cerebral and systemic hemodynamics. We have recently demonstrated, using PCMRI, that induced hypertension with NE, raising MAP by approximately 20% in healthy individuals, paradoxically reduced CBF [22]. This finding challenges the assumption that MAP is a reliable surrogate for cerebral perfusion and provides strong motivation to extend investigation to patients under anesthesia and in critical illness.

This protocol describes two parallel studies applying identical MRI-based methodology in combination with continuous invasive blood pressure monitoring and NIRS: MAP-ANE in elective anesthesia patients and MAP-SAH in sedated ICU patients with aSAH. The objective is to characterize MAP–CBF relationships under these two conditions, test the hypothesis that blood pressure augmentation does not increase CBF, and explore interactions between the cerebral and systemic circulation. A further aim is to assess the validity of NIRS as a surrogate marker for CBF changes across different blood pressure levels.

## Materials and methods

### Study design

**Fig 1:**
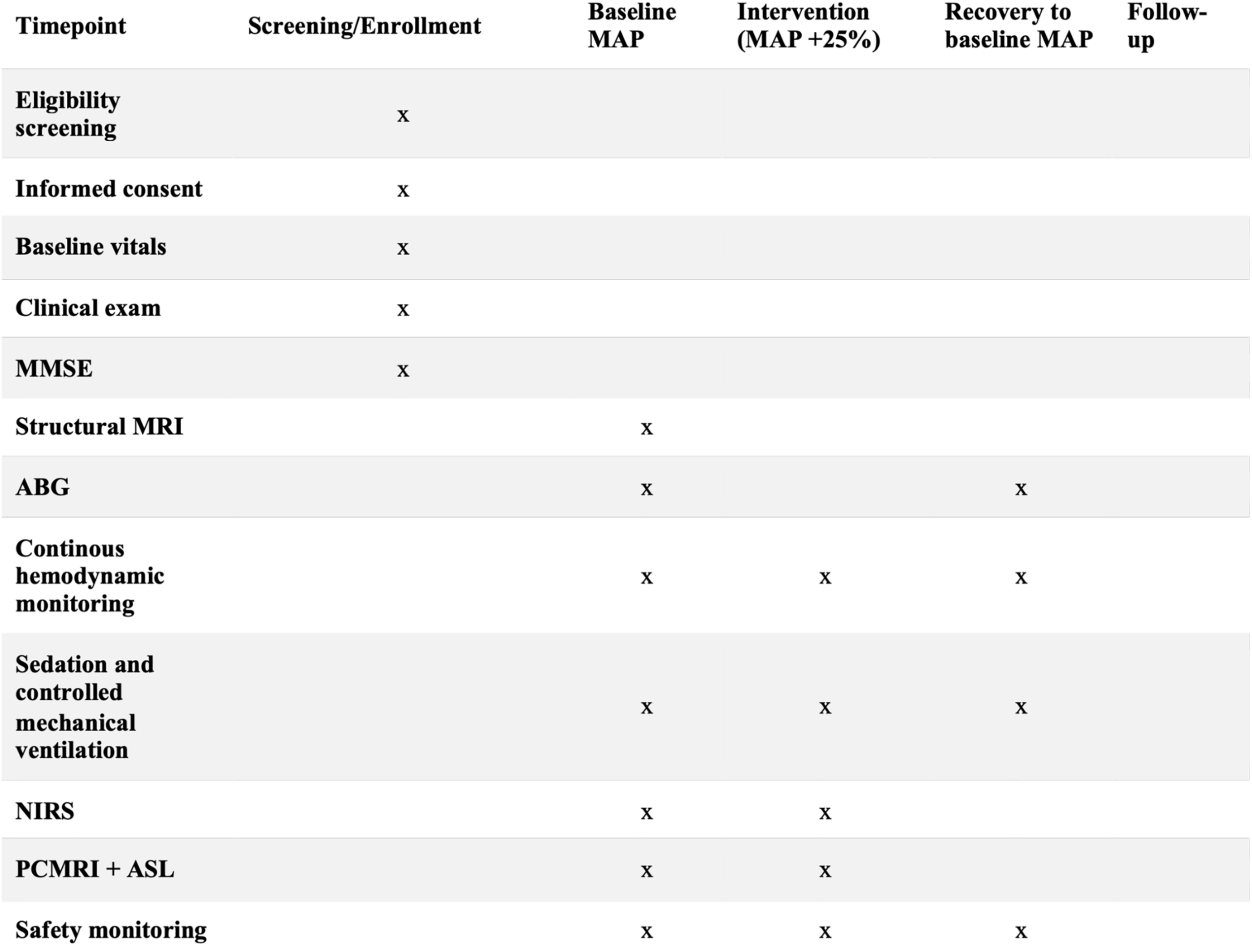
SPIRIT schedule of enrollment, interventions and assessments for MAP-ANE (elective general anesthesia study population). MAP, mean arterial pressure; MMSE, Mini Mental State Examination; ABG, arterial blood gas; NIRS, near-infrared spectroscopy; PCMRI, phase-contrast magnetic resonance imaging; ASL, arterial spin labeling.

**Fig 2:**
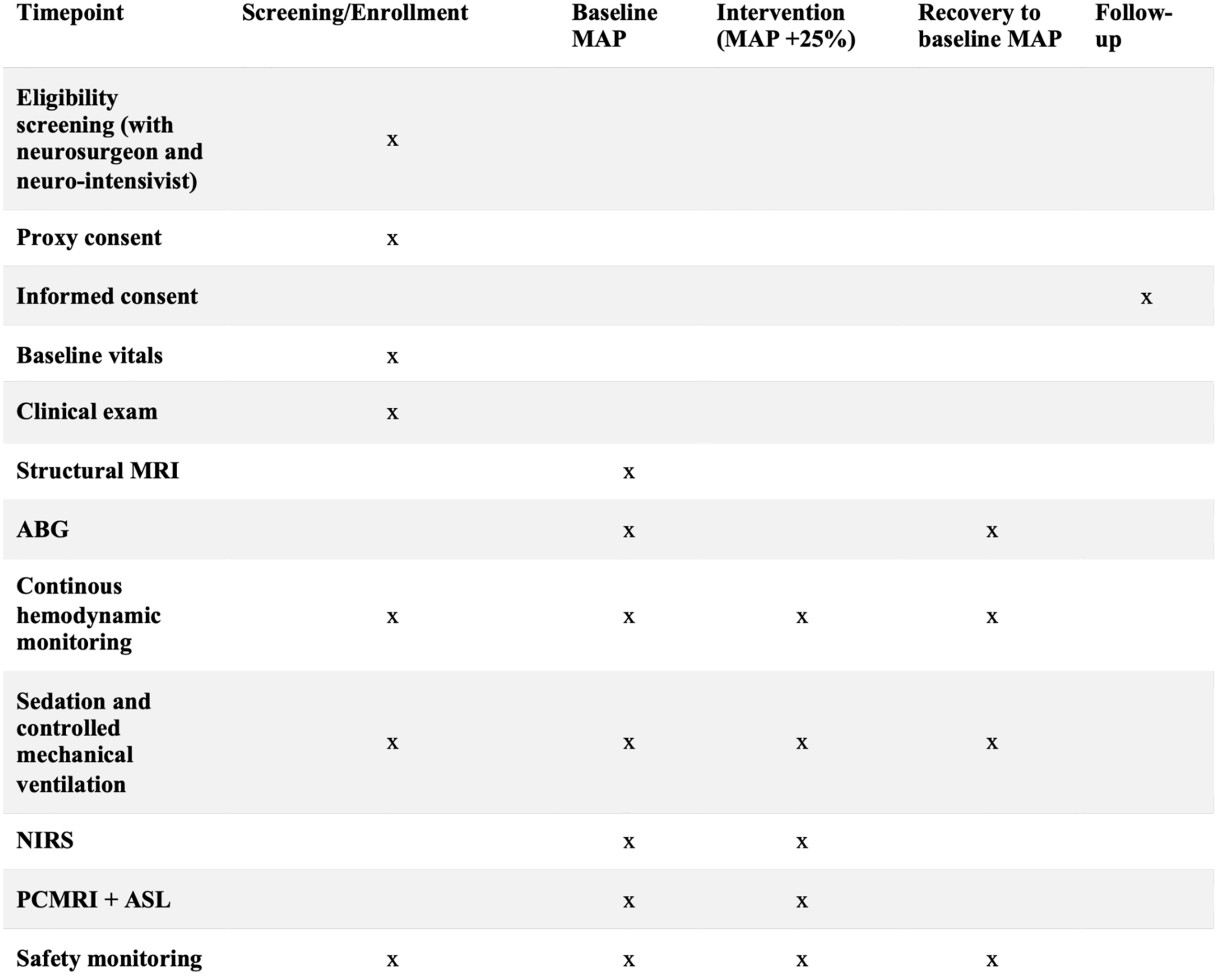
SPIRIT schedule of enrollment, interventions and assessment for MAP-SAH (sedated ICU study population). MAP, mean arterial pressure; ABG, arterial blood gas; NIRS, near-infrared spectroscopy; PCMRI, phase-contrast magnetic resonance imaging; ASL, arterial spin labeling.

**Fig 3:**
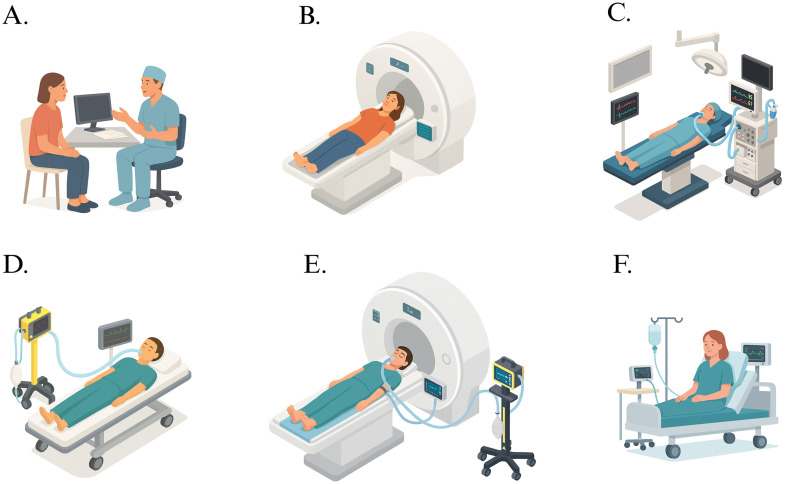
Graphical study flow for MAP-ANE (elective general anesthesia study population). A. Screening and informed consent. B. Structural MRI and baseline PCMRI/ASL while awake without MAP increase. C. Ear, Nose and Throat surgery under general anesthesia followed by NIRS monitoring during a ~ 25% MAP increase with norepinephrine as a safety check to ensure tolerance without arrhythmias before MRI transport. D. Transport to MRI. E. PCMRI and ASL at baseline MAP and during a ~ 25% MAP increase with norepinephrine. F. Emergence from anesthesia and recovery.

**Fig 4:**
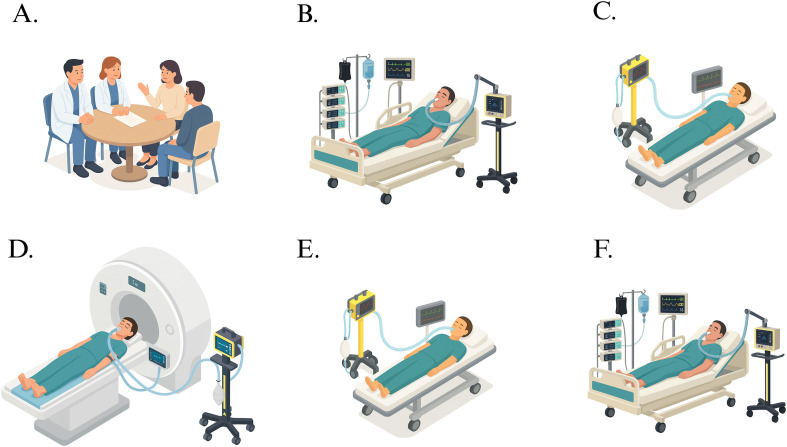
Graphical study flow for MAP-SAH (sedated ICU study population). A. Screening and inclusion with approval from both the ICU physician and the responsible neurosurgeon, and consent from next of kin. B. ICU care under sedation with NIRS monitoring during a ~ 25% MAP increase with norepinephrine as a safety check to ensure tolerance without arrhythmias before MRI transport. C. Transport to MRI. D. Structural MRI followed by PCMRI and ASL at baseline MAP and during a ~ 25% MAP increase with norepinephrine. E. Transport back to the ICU. F. Continued neurointensive care and monitoring.

This study protocol follows the SPIRIT guidelines [23] and describes two non-randomized, prospective interventional studies with separate schedules for MAP-ANE and MAP-SAH (Fig 1 and 2). Graphical study flow diagrams (Fig 3 and 4) illustrate the process from screening and inclusion to completion of the intervention and, for ICU patients, return to intensive care. The design is within-subject and paired, with each participant serving as their own control by undergoing measurements at baseline MAP and after a standardized ~25% increase in MAP. No blinding is applied during the intervention. Participant recruitment for MAP-SAH began on October 6, 2023, and for MAP-ANE on December 6, 2024. Recruitment and data collection for MAP-ANE are expected to be completed by the end of 2026, whereas MAP-SAH is planned to continue until October 2030, with follow-up concluding at the same time. Data analysis and dissemination of results are planned during 2027 for MAP-ANE and following completion of MAP-SAH. No study results have yet been generated.

### Study setting

The study is conducted at Umeå University Hospital, Umeå, Sweden. Documentation of all anesthetic and hemodynamic data is carried out in the hospital’s electronic patient data management system (PDMS) Metavision, version 6.8 (iMDsoft, Tel Aviv, Israel). Study-specific variables not captured in PDMS are recorded in paper-based case report forms (CRFs), which are securely stored in locked cabinets at the study site. MAP-ANE is conducted in the operating theatre setting during elective Ear, Nose and Throat surgery, and MAP-SAH is performed in the level 3 ICU (10 beds, with approximately 25% neurointensive care bed occupancy) among sedated patients with aSAH. The MRI examinations are carried out in the hospital’s 3T MRI unit, located on the same floor and equipped to enable examinations under general anesthesia.

### Recruitment and consent

Participants for the MAP-ANE and MAP-SAH studies are recruited at Umeå University Hospital, Umeå, Sweden, from patients undergoing elective general anesthesia and from sedated ICU patients with aSAH. The inclusion and exclusion criteria for both study populations are summarized in Table 1. Elective patients (MAP-ANE) will be enrolled preoperatively after providing written informed consent. For ICU patients (MAP-SAH), informed consent will be obtained from next of kin and subsequently confirmed by the patient if they regain decision-making capacity.

**Table 1:** Inclusion and exclusion criteria for the MAP-ANE (elective general anesthesia) and MAP-SAH (sedated ICU) study populations.

MAP-ANE	MAP-SAH
**Inclusion**	**Inclusion**
Age 50–80 years	Confirmed aSAH treated with endovascular coiling or surgical clipping
Elective Ear, Nose and Throat surgery under general anesthesia	Ongoing sedation with mechanical ventilation
Oral and written informed consent	Invasive arterial monitoring
Mini-Mental State Examination (MMSE) >23	Clinical stability sufficient to undergo MRI
ASA classification I–III	Informed consent obtained from next of kin
Completed cardiovascular and neurological screening	Exclusion
Structural MRI prior to intervention	MRI contraindications
**Exclusion**	Medical instability precluding transport to MRI
MRI contraindications (implanted devices, ferromagnetic metal/implants)	Tachycardia or bradycardia (heart rate >100 or <50 beats/min)
BMI > 30	High oxygen requirements (FiO2 > 60%)
	COPD stage 3–4
	BMI > 30
	Patient does not subsequently consent

aSAH, aneurysmal subarachnoid hemorrhage; ASA, American Society of Anesthesiologists; MRI, magnetic resonance imaging; ICU, intensive care unit; FiO_2_, fraction of inspired oxygen; COPD, chronic obstructive pulmonary disease; BMI, body mass index.

### Intervention

Blood pressure and vital signs will be monitored with MRI-compatible equipment Philips Expression MR400 (Philips Healthcare, Best, The Netherlands) during scanning and Philips IntelliVue X2 (Philips Healthcare, Best, The Netherlands) in the operating room and ICU. Invasive continuous blood pressure monitoring is achieved via a radial artery catheter with a fluid-filled line and Xtrans® pressure transducer (CODAN TRIPLUS AB, Kungsbacka, Sweden). Electrocardiogram, pulse oximetry, systolic and diastolic pressure, MAP, and end-tidal carbon dioxide (EtCO₂) are continuously recorded using the respective patient monitoring systems, with all data exported to the PDMS. Administered drugs are entered manually into the PDMS. Arterial blood gases will be sampled immediately before and after the intervention and analyzed using ABL90 FLEX PLUS (Radiometer Medical ApS, Brønshøj, Denmark). For MAP-SAH patients, ICP is continuously monitored in the ICU using an intraparenchymal or intraventricular catheter, with values exported to the PDMS.

As a safety measure, blood pressure elevation is carried out prior to transport to the MRI suite in the operating room (MAP-ANE) or ICU (MAP-SAH). During this manipulation, NIRS measurements are obtained with the INVOS™ 5100C (Medtronic, Minneapolis, MN, USA). Baseline MAP is defined by the attending anesthesiologist according to clinical routines, typically 65–70 mmHg. If NE infusion is already required to maintain this level, it is continued at the same dose and documented as the baseline NE dose. For the blood pressure elevation procedure, NE infusion is titrated stepwise every 2 minutes by 0.01–0.04 μg·kg⁻¹·min⁻¹ until a 25% MAP increase or a systolic pressure limit of 160 mmHg in MAP-ANE and 200 mmHg in MAP-SAH is reached. MRI scans will be acquired at both baseline and elevated MAP. NE (Sintetica, Mendrisio, Switzerland) is prepared as a 40 µg/mL solution and administered via a Braun Perfusor Space pump (B. Braun, Melsungen, Germany), with Plasmalyte (Baxter, Deerfield, IL, USA) delivered by Braun Infusomat Space (B. Braun, Melsungen, Germany) as the carrier. The intervention lasts approximately 90 minutes. All participants are monitored by an anesthesiologist, and NE infusion is discontinued at any sign of adverse response.

### Anesthesia management

During MRI acquisition in both MAP-ANE and MAP-SAH, anesthesia is maintained with propofol 20 mg/mL (Baxter, Deerfield, IL, USA) administered as a continuous infusion at 4–8 mg·kg ⁻ ¹·h ⁻ ¹, titrated as needed. An intravenous bolus dose of fentanyl 150–200 μg (B. Braun, Melsungen, Germany) is administered immediately before transfer into the MRI scanner. The depth of anesthesia is assessed continuously by the attending anesthesiologist. Sedation and mechanical ventilation are maintained throughout transport from the operating room (MAP-ANE) or ICU (MAP-SAH) to the MRI suite and during the imaging procedure.

### Outcomes

The primary outcome is the change in total CBF between baseline and elevated MAP, thereby directly testing whether induced hypertension increases CBF. Secondary outcomes include additional perfusion measurement with ASL, characterization of the slope of the MAP–CBF relationship, and analysis of associations between systemic and cerebral hemodynamic parameters, such as cardiac output (CO), systemic vascular resistance (SVR), cerebrovascular resistance (CVR), and arterial compliance. Further, changes in rSO_2_ relative to CBF will be analyzed to evaluate its validity as a surrogate marker. Safety parameters, including arrhythmias, bradycardia, or other adverse responses during NE infusion, are monitored continuously. Events severe enough to require interruption of the procedure and discontinuation of NE infusion are documented in the CRF.

### MRI acquisition

Brain MRI screening will include T1- and T2-weighted, T2-FLAIR, and time-of-flight angiography sequences. During the study procedure, T1-weighted 3D images are acquired for calculating brain volume (magnetization prepared rapid gradient echo imaging with repetition time/echo time/flip angle of 10.3 ms/4.9 ms/8°). Brain volumes are automatically segmented using FreeSurfer (Athinoula A. Martinos Center for Biomedical Imaging, Massachusetts General Hospital, Boston, MA, USA). All scans performed during the study procedure will be performed on a Philips Ingenia 3T system (Philips Healthcare, Best, The Netherlands) with a 20-channel head–neck coil. A first PCMRI plane is placed at the C2–C3 level of the neck, with the following parameters: retrospective gating using peripheral pulse recording, heart phases = 32, acquired voxel size = 1 × 1 mm with 3-mm slice thickness, repetition time/echo time = 9.2 ms/5.5 ms, flip angle = 10°, and velocity encoding = 80 cm/s. A second PCMRI plane is placed perpendicular to the ascending aorta, thus also transecting the descending aorta, with the following parameters: retrospective gating using peripheral pulse recording, heart phases = 32, acquired voxel size = 2.5 × 2.5 mm with 8-mm slice thickness, repetition time/echo time = 4.2 ms/2.6 ms, flip angle = 10°, and velocity encoding = 150 cm/s. Flow measurements will be performed using Segment version 3.2 R9074 (Medviso AB, Lund, Sweden) [24]. For each voxel of the phase image, velocity (cm/s) is calculated and averaged over the region of interest (ROI). Blood flow rate (mL/min) is then obtained by multiplying the average velocity during the cardiac cycle with the cross-sectional ROI area. The images are manually inspected for signs of aliasing or motion artefacts, and any aliasing is corrected using the built-in feature in Segment. For the C2–C3 planes, vessel lumen segmentation is either manually defined and kept constant across the cardiac cycle or performed with the semiautomatic algorithm, which tracks the vessel throughout the cycle. For the aortic planes, segmentation is performed using the semiautomatic algorithm. Cerebral perfusion will be acquired using pseudo-continuous arterial spin labeling (pcASL), with a single-shot gradient-echo EPI readout. The sequence will be acquired with TR/TE = 4139/13 ms, flip angle = 90°, field of view = 240 × 240 × 150 mm³, and an acquisition matrix of 86 × 68, yielding an acquired voxel size of 2.73 × 3.75 × 5.0 mm³ (reconstructed to 1.88 × 1.88 × 5.0 mm³). 30 transverse slices (0 mm gap) will be collected with parallel imaging (SENSE factor 2.3, AP direction). Labeling will be applied below the imaging slab using a pcASL scheme, followed by a post-labeling delay of 2000 ms. Thirty dynamic control/label pairs will be obtained, with a total scan duration of 7 min 2 s. Fat suppression will be performed using SPIR. All images will be co-registered to the high-resolution T1-weighted anatomical scan. Perfusion maps are quantified using the Oxford ASL script in the BASIL toolbox (FSL, Oxford Centre for Integrative Neuroimaging) [25], following consensus recommendations [26]. Here, voxel-wise control–label differences will be converted to perfusion estimates using a model of MR signal evolution based on acquisition parameters and blood and tissue relaxation properties.

### Hemodynamic characteristics

Total CBF is calculated as the sum of blood flow rates (mL/min) in the left and right internal carotid and vertebral arteries. CO is represented by ascending aorta flow rate, stroke volume is derived from CO divided by heart rate. The flows in the external carotid arteries are summed and interpreted as a characterization of peripheral vascular flow. CVR is calculated as (MAP – ICP)/CBF. SVR is calculated as ([MAP – central venous pressure (CVP)]/CO) × 80. CVP is assumed to be 10 mmHg in both groups [27]. ICP is assumed to be 10 mmHg in MAP-ANE [28], whereas in MAP-SAH the recorded mean ICP from the hour preceding the intervention is used. Flow areas are calculated using ROI for all vessels. Arterial compliance will be calculated from PCMRI-derived flow waveforms and simultaneous arterial pressure data as previously described [29]. An overview of measured and derived variables is provided in Table 2.

**Table 2:** An overview of all measured and derived variables.

Variable	Source/method	Unit
**Measured variables**		
MAP, SAP, DAP	Invasive radial artery catheter	mmHg
Heart rate (HR)	ECG, MRI	beats/min
Oxygen saturation (SpO_2_)	Pulse oximetry	%
End-tidal CO₂ (EtCO_2_)	MR400	kPa
Arterial blood gases	ABL90 FLEX PLUS	kPa and mmol/L
Regional cerebral oxygenation (rSO_2_)	NIRS (INVOS™ 5100C)	%
ICA, VA, ECA blood flow	PCMRI (C2–C3 planes)	mL/min
Aortic blood flow/ cardiac output (CO)	PCMRI (ascending aorta plane)	mL/min
Regional perfusion	ASL (pcASL sequences)	mL/100 g/min
Intracranial pressure (ICP)	Intraparenchymal or intraventricular catheter	mmHg
**Calculated variables**		
Stroke volume	CO ÷ HR	mL/beat
Total CBF	Sum of bilateral ICA + VA flows	mL/min
External carotid artery flow	Summed ECA flows	mL/min
Cerebrovascular resistance (CVR)	(MAP – ICP) ÷ CBF	mmHg· mL^-1^· min^-1^
Systemic vascular resistance (SVR)	([MAP – CVP] ÷ CO) × 80	dyn· secs· cm^-5^
Brain volume	FreeSurfer automated segmentation of T1-weighted sequence	mL
Arterial compliance	Windkessel model, ratio of pulsatile volume to pressure	mL/mmHg

MAP, mean arterial pressure; SAP, systolic arterial pressure; DAP, diastolic arterial pressure; NIRS, near-infrared spectroscopy; ICA, internal carotid artery; VA, vertebral artery; ECA, external carotid artery; CO, cardiac output; PCMRI, phase-contrast magnetic resonance imaging; ASL, arterial spin labeling; CBF, cerebral blood flow; ICP, intracranial pressure; CVP, central venous pressure.

### Sample size

A non-inferiority margin of +15% is specified as the minimal clinically important increase in total CBF. This threshold exceeds expected measurement repeatability and short-term biological variability for PCMRI [30] and remains conservative relative to prior transcranial Doppler data in traumatic brain injury and critically ill patients reporting ~20% increases in cerebral blood flow velocity at ~30% MAP elevation [15]. To minimize type II error, the study is powered at 95% (two-sided α = 0.05). Missing a true increase in global CBF would have direct clinical implications, as it could falsely support withholding potentially beneficial MAP augmentation. Sample size estimation is based on the standard deviation of CBF change observed in prior experimental studies of healthy volunteers. To account for greater expected heterogeneity in anesthesia and ICU patients, this standard deviation was conservatively doubled. Under these assumptions, the required sample size is 18 analyzable paired datasets per cohort. Allowing for ~10% attrition, we plan to enroll 20 participants per study population (MAP-ANE and MAP-SAH).

### Statistical methods

All analyses will be performed using SPSS (IBM Corp., Armonk, NY, USA). Given the relatively small sample size, assumptions of normality cannot be reliably validated; therefore, non-parametric tests will be used. Descriptive statistics will be reported as medians with interquartile range unless otherwise specified. Changes in flow, pressure, resistance, arterial compliance, and vessel area between baseline and elevated MAP will be analyzed using the Wilcoxon signed-rank test. Correlations between systemic and cerebral hemodynamic variables will be explored using Spearman’s rank correlation. A two-sided p-value <0.05 will be considered statistically significant.

### Ethical considerations

The study protocols for both MAP-ANE and MAP-SAH were reviewed and approved by the Swedish Ethical Review Authority (*Etikprövningsmyndigheten*), the national institutional review board for human research in Sweden. For MAP-ANE, the main approval (approval number 2024-00251-01) was granted on June 3, 2024, and an amendment (approval number 2024-05508-02) was approved on October 2, 2024. For MAP-SAH, the main approval (approval number 2022-06754-01) was granted on February 21, 2023, with subsequent amendments approved on March 27, 2023 (2023-01491-02) and June 10, 2024 (2024-03315-02). Both studies are conducted in accordance with the 1964 Declaration of Helsinki and its later amendments. All participants in MAP-ANE provide written informed consent prior to enrollment. For MAP-SAH, informed consent is obtained from next of kin and subsequently confirmed by the patient if possible. Participation is voluntary, and no financial compensation is provided. Both studies are registered at ClinicalTrials.gov (MAP-ANE: NCT06855407; MAP-SAH: NCT06033378). The authors confirm that all ongoing and related trials for this intervention are registered. Data are pseudonymized and stored on secure hospital servers in compliance with the General Data Protection Regulation (GDPR). Results will be disseminated through peer-reviewed publications and conference presentations regardless of outcome.

## Discussion

This study protocol addresses the uncertainty surrounding the relationship between MAP and CBF during anesthesia and critical illness, with a focus on NE-induced blood pressure elevation. By applying standardized methodology across two distinct but related patient populations, elective anesthesia patients and sedated aSAH patients, we will be able to compare MAP–CBF relationships under different physiological conditions. The use of PCMRI and ASL allows direct and quantitative assessment of global cerebral blood flow, which overcomes the limitations of surrogate methods such as NIRS.

A major strength of the study is its design, combining invasive hemodynamic monitoring with advanced MRI-based flow measurements. This multimodal approach enables us to test the hypothesis that blood pressure augmentation with NE does not increase CBF, and to assess whether NIRS can serve as a valid bedside proxy for CBF changes. The parallel investigation of two study populations enhances generalizability, as one group represents controlled perioperative conditions while the other reflects neurocritical care after aSAH.

Several challenges and limitations must be acknowledged. MRI measurements are not feasible at the bedside, which may limit clinical applicability. However, the purpose of this study is not to provide a bedside monitoring tool, but to investigate fundamental pathophysiological mechanisms linking MAP and CBF under anesthesia and in critical illness. By establishing such principles with quantitative MRI, subsequent research can evaluate whether surrogate measures such as NIRS may reliably capture comparable information in the clinical setting.

Transport of sedated and ventilated ICU patients carries inherent risks, although these will be mitigated through rigorous safety procedures and continuous supervision by an anesthesiologist. Sample size is relatively modest, which restricts subgroup analyses and may limit statistical power for secondary outcomes. Finally, while inclusion of both study populations enhances the clinical relevance of the results, generalization beyond these groups should be made cautiously.

Despite these limitations, the expected results have the potential to provide novel insights into cerebral autoregulation and the hemodynamic effects of induced hypertension. Clarifying the relationship between MAP and CBF is clinically relevant in both anesthesia and neurocritical care, where blood pressure management is central to optimizing cerebral perfusion and preventing secondary injury.

### Trial status

Recruitment and data collection are ongoing at the time of manuscript submission.

## Supporting information

S1 Checklist SPIRIT 2025-checklist. (DOCX)

## Abbreviations

aSAH: aneurysmal subarachnoid hemorrhage
ASA: American Society of Anesthesiologists
ASL: arterial spin labeling
MAP-ANE: patients under general anesthesia study population
MAP-SAH: sedated ICU patients with aSAH study population
CBF: cerebral blood flow
CPP: cerebral perfusion pressure
CO: cardiac output
CVR: cerebrovascular resistance
DCI: delayed cerebral ischemia
EtCO_2_: end-tidal carbon dioxide
ICP: intracranial pressure
MAP: mean arterial pressure
MRI: magnetic resonance imaging
NIRS: near-infrared spectroscopy
PCMRI: phase-contrast magnetic resonance imaging
rSO_2_: regional cerebral oxygen saturation
SVR: systemic vascular resistance
TCD: transcranial Doppler.
